# European Safety Analysis of mRNA and Viral Vector COVID-19 Vaccines on Glucose Metabolism Events

**DOI:** 10.3390/ph15060677

**Published:** 2022-05-27

**Authors:** Gabriella di Mauro, Annamaria Mascolo, Miriam Longo, Maria Ida Maiorino, Lorenzo Scappaticcio, Giuseppe Bellastella, Katherine Esposito, Annalisa Capuano

**Affiliations:** 1 Campania Regional Centre for Pharmacovigilance and Pharmacoepidemiology, 80138 Naples, Italy; gabriella.dimauro@unicampania.it (G.d.M.); annalisa.capuano@unicampania.it (A.C.); 2 Department of Experimental Medicine, Section of Pharmacology “L. Donatelli”, University of Campania “Luigi Vanvitelli”, 80138 Naples, Italy; 3 Department of Advanced Medical and Surgical Sciences, University of Campania “Luigi Vanvitelli”, 80138 Naples, Italy; miriam.longo@unicampania.it (M.L.); mariaida.maiorino@unicampania.it (M.I.M.); lorenzo.scappaticcio@unicampania.it (L.S.); giuseppe.bellastella@unicampania.it (G.B.); katherine.esposito@unicampania.it (K.E.); 4 Division of Endocrinology and Metabolic Diseases, University of Campania “Luigi Vanvitelli”, 80138 Naples, Italy

**Keywords:** COVID-19 vaccines, diabetes mellitus, glucose metabolism events, safety, Europe

## Abstract

Few data have been published on the effects of impaired glucose metabolism induced by COVID-19 vaccines. We decided to perform a study to describe Individual Case Safety Reports (ICSRs) of impaired glucose metabolism events reported in the European database (Eudravigilance, EV). ICSRs were retrieved from the online website of Eudravigilance. The reporting odds ratios (ROR) were computed to assess the reporting frequency for COVID-19 mRNA vaccines compared to COVID-19 viral vector-based vaccines. A total of 3917 ICSRs with a COVID-19 vaccine suspected were retrieved, with a total of 4275 impaired glucose metabolism events. Overall, the most reported events were related to “high glucose levels” (2012; 47.06%). The mRNA vaccines were associated with an increased reporting frequency of “type 1 diabetes mellitus” (ROR 1.86; 95% CI 1.33–2.60), “type 2 diabetes mellitus” (ROR 1.58; 95% CI 1.03–2.42), “high glucose levels” (ROR 1.16; 95% CI 1.06–1.27), “diabetes mellitus inadequate control” (ROR 1.63; 95% CI 1.25–2.11), and “hypoglycemia” (ROR 1.62; 95% CI 1.41–1.86) compared to viral vector-based vaccines. mRNA COVID-19 vaccines were associated with an increased reporting frequency of alterations of glucose homeostasis compared to viral-vector COVID-19 vaccines. Clinicians should be aware of these events to better manage glycemic perturbations. Larger nationwide studies are warranted to verify these findings.

## 1. Introduction

Since the beginning of the Coronavirus Disease 2019 (COVID-19) outbreak, several efforts have been made to develop effective therapeutic prevention strategies [1]. It is well-known that COVID-19 is a complex disease characterized by several clinical phases of progression, affecting many organs apart from the respiratory tract [2,3]. This disease has shown worse prognosis both in patients with type 1 and type 2 diabetes mellitus [4]. Indeed, diabetes mellitus is considered a contributing risk factor to the severity and mortality of COVID-19 [5]. Based on these considerations, vaccination for COVID-19 is a priority for this subpopulation [6]. To date, five vaccines for the prevention of COVID-19 have been approved by the European Medicine Agency (EMA) with a conditional marketing authorization [7]. Specifically, the first vaccine authorized was produced by Pfizer–BioNTech, followed by the one manufactured by Moderna, which both share the same innovative mRNA vaccine technology. Later, two viral vector-based vaccines manufactured by Oxford–AstraZeneca and Johnson & Johnson (Janssen) were approved. Recently, a protein-based vaccine developed by Novavax was also approved. Since their approval, data on the safety profile have been published, but few have been related to the occurrence of impaired glucose metabolism events. Case series showed a link between such events and COVID-19 vaccination in both patients with and without diabetes mellitus [8,9,10,11,12,13]. Based on this gap in knowledge, we decided to perform a study to describe Individual Case Safety Reports (ICSRs) of impaired glucose metabolism events reported in the European database (Eudravigilance, EV) and to assess the reporting frequency of COVID-19 vaccines.

## 2. Results

During the study period, we identified a total of 3917 ICSRs reporting a COVID-19 vaccine as suspected, and at least one event of impaired glucose metabolism. Specifically, 2027 (51.75%) ICSRs referred to the Pfizer–BioNTech vaccine, 586 (14.96%) the Moderna vaccine, 1163 (29.70%) the Oxford–AstraZeneca vaccine, and 141 (3.59%) the Janssen vaccine. Six ICSRs (0.15%) reported instead a heterologous vaccination, defined by the presence of two different COVID-19 vaccine products as suspected vaccines. In particular, three ICSRs reported Oxford–AstraZeneca/Pfizer–BioNTech vaccines, two Pfizer–BioNTech/Moderna vaccines and one with Oxford–AstraZeneca/Moderna vaccines. The age group most reported was 18–64 years for all types of COVID-19 vaccines. ICSRs mostly referred to female patients for Pfizer–BioNTech (*n* = 1194; 58.91%), Moderna (*n* = 331; 56.48%), and Oxford–AstraZeneca (*n* = 699; 60.10%) vaccines, while a roughly equal gender distribution was observed for the Janssen vaccine (48.23% female vs. 51.06% male). In 269 (6.87%) and 61 (1.56%) ICSRs, respectively, age and gender were not reported. The main primary source was healthcare professionals (HCP) for ICSRs with Moderna (*n* = 411; 70.14%) and Janssen (*n* = 81; 57.45%) vaccines, while non-HCP for Pfizer–BioNTech (*n* = 1177; 58.07%) and Oxford–AstraZeneca (*n* = 799; 68.70%) vaccines. The majority of ICSRs with Pfizer–BioNTech vaccine (*n* =1170; 57.72%) were from the European Economic Area (EEA), with the remaining from Non-EEA. In most ICSRs, concomitant antidiabetic drugs were not reported for all types of vaccines (72.07%). Table 1 and Table 2 report all characteristics of ICSRs by type of COVID-19 vaccine.

### 2.1. Impaired Glucose Metabolism Events

**Figure 1:**
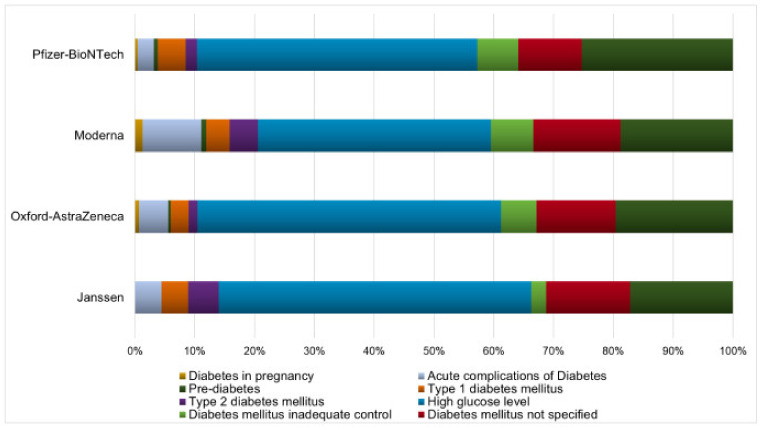
Distribution of impaired glucose metabolism events. Percentages of reporting impaired glucose metabolism events for groups (“diabetes in pregnancy”, “acute complications of diabetes”, “pre-diabetes”, “type 1 diabetes mellitus”, “type 2 diabetes mellitus”, “high glucose levels”, “diabetes mellitus inadequate control”, “diabetes mellitus not specified”, and “hypoglycaemia”) and type of COVID-19 vaccine (Pfizer-BioNTech, Moderna, Oxford–AstraZeneca, and Janssen vaccines) reported in Eudravigilance from 1 January 2021 to 11 December 2021.

**Figure 2:**
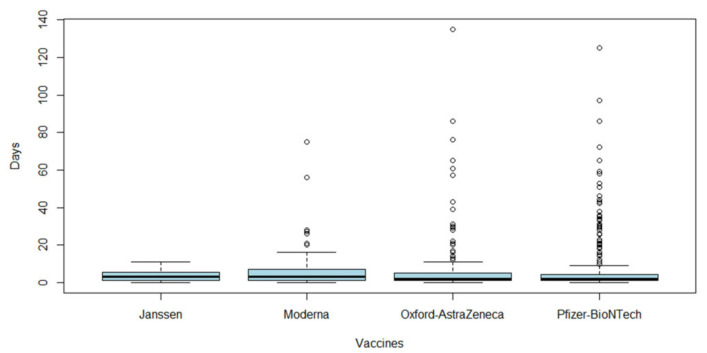
Time for impaired glucose metabolism events. Boxplots of duration of all impaired glucose metabolism events, expressed in days, for each COVID-19 vaccine (Pfizer–BioNTech, Moderna, Oxford–AstraZeneca, or Janssen) and retrieved from Eudravigilance for the period 1 January 2021 to 11 December 2021.

From 3917 ICSRs, we observed a total of 4275 impaired glucose metabolism events (1.09 adverse events per ICSR) since more than one adverse event could be reported in each ICSR. Specifically, 2194 (51.32%) events for Pfizer–BioNTech vaccine, 1286 (30.08%) for Oxford–AstraZeneca vaccine, 638 (14.92%) for Moderna vaccine, and 157 (3.68%) for Janssen vaccine. The monthly trend of impaired glucose metabolism events is shown in Figure S1. The total number of adverse events for each type of vaccine is reported in Table S3. Overall, the most-reported events belong to the group “high glucose levels” (*n* = 2012; 47.06%), followed by “hypoglycaemia” (*n* = 954; 22.32%), “diabetes mellitus not specified” (*n* = 518; 12.12%), “diabetes mellitus inadequate control” (*n* = 275; 6.43%), “acute complications of diabetes” (*n* = 193; 4.51%), “type 1 diabetes mellitus” (*n* = 173; 4.05%), “type 2 diabetes mellitus” (*n* = 98; 2.29%), “diabetes in pregnancy” (*n* = 27; 0.63%), and “pre-diabetes” (*n* = 24; 0.56%). The distribution of all event groups and preferred terms (PTs) for each type of COVID-19 vaccine is reported in Figure 1 and Table S4, respectively. Adverse events were mostly classified as serious (*n* = 2694; 63.02%). Specifically, the three most-reported seriousness criteria were: other medically important conditions (*n* = 1429; 33.43%), caused or prolonged hospitalization (*n* = 642; 15.02%), and life-threatening (*n* = 300; 7.02%). The outcome was favorable for most events (*n* = 1851; 43.30%). In 2.71% of events, the outcome was fatal. Seriousness and outcome criteria for COVID-19 vaccines are presented in Table 3 and Table 4. The duration of adverse events was reported in 808 ICSRs, with a median of 2.00 days (interquartile range, IQR: 4.25–1.00) for Pfizer–BioNTech vaccine, 3.00 days (IQR: 7.00–1.00) for Moderna, 2.00 days (IQR: 5.00–1.00) for Oxford–AstraZeneca and 3.00 days (IQR: 4.75–1.00) for Janssen (Figure 2).

### 2.2. ROR in the Main Analysis (All ICSRs)

**Figure 3:**
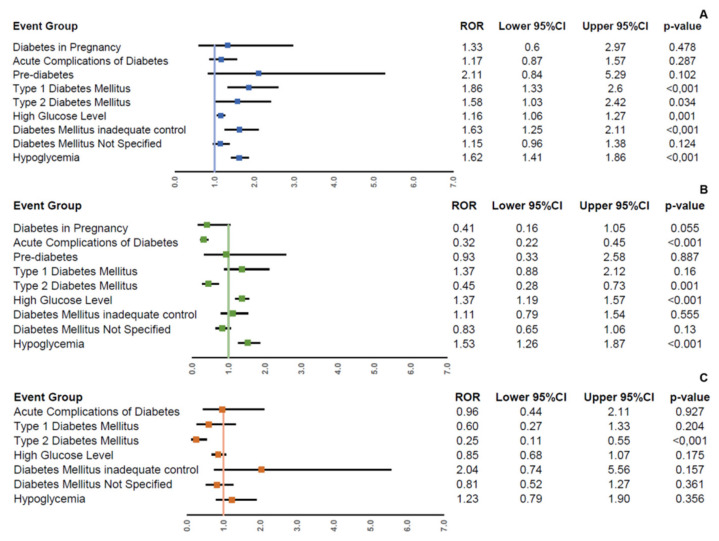
Reporting frequency of impaired glucose metabolism groups among COVID-19 vaccines. Reporting odds ratios (RORs) and their 95% confidence interval (95% CI) of impaired glucose metabolism groups comparing COVID-19 mRNA vaccines with COVID-19 viral vector-based vaccines (**A**), Pfizer–BioNTech vaccine with Moderna vaccine (**B**), and Oxford–AstraZeneca vaccine with Janssen vaccine (**C**) in all Individual Case Safety Reports (ICSRs) reported in Eudravigilance from 1 January 2021 to 11 December 2021.

The mRNA vaccines were associated with an increased reporting frequency of “type 1 diabetes mellitus” (reporting odds ratio, ROR 1.86; 95% confidence interval, 95% CI 1.33–2.60), “type 2 diabetes mellitus” (ROR 1.58; 95% CI 1.03–2.42), “high glucose levels” (ROR 1.16; 95% CI 1.06–1.27), “diabetes mellitus inadequate control” (ROR 1.63; 95% CI 1.25–2.11), and “hypoglycemia” (ROR 1.62; 95% CI 1.41–1.86) compared to viral vector-based vaccines (Figure 3A). In the comparison between mRNA vaccines, Pfizer–BioNTech vaccine was associated with an increased reporting frequency of “high glucose levels” (ROR 1.37; 95% CI 1.19–1.57) and “hypoglycemia” (ROR 1.53; 95% CI 1.26–1.87) compared to Moderna vaccine (Figure 3B). Finally, no higher reporting frequency was observed for Oxford–AstraZeneca vaccine compared to Janssen vaccine for all impaired glucose metabolism groups (Figure 3C).

### 2.3. ROR in Sensitivity Analyses (ICSRs with or without Concomitant Antidiabetic Agents)

In the analysis considering only ICSRs with concomitant anti-diabetic agents, mRNA vaccines were associated with an increased reporting frequency of “hypoglycemia” (ROR 1.70; 95% CI 1.30–2.22) compared to viral vector-based vaccines (Figure S2A). In the comparison between mRNA vaccines, Pfizer–BioNTech vaccine was associated with an increased reporting frequency of “high glucose levels” (ROR 2.19; 95% CI 1.68–2.85), “diabetes mellitus inadequate control” (ROR 2.65; 95% CI 1.35–5.21), and “hypoglycemia” (ROR 2.82; 95% CI 1.88–4.25) compared to Moderna vaccine (Figure S2B). Finally, Oxford–AstraZeneca vaccine was associated with an increased reporting frequency of “high glucose levels” (ROR 1.53; 95% CI 1.02–2.28) and “hypoglycemia” (ROR 2.33; 95% CI 0.85–6.38) compared with Janssen vaccine (Figure S2C). In the analysis considering instead ICSRs without concomitant anti-diabetic agents, mRNA vaccines were associated with an increased reporting frequency of “type 1 diabetes mellitus” (ROR 2.12; 95% CI 1.47–3.06), “high glucose levels” (ROR 1.53; 95% CI 1.36–1.72), “diabetes mellitus inadequate control” (ROR 1.75; 95% CI 1.26–2.42), “diabetes mellitus not specified” (ROR 1.32; 95% CI 1.08–1.62), and “hypoglycemia” (ROR 1.63; 95% CI 1.38–1.92) compared to viral vector-based vaccines (Figure S3A). Moreover, Pfizer–BioNTech vaccine was associated with an increased reporting frequency of “high glucose levels” (ROR 1.32; 95% CI 1.12–1.56) and “hypoglycemia” (ROR 1.42; 95% CI 1.13–1.78) compared to Moderna vaccine (Figure S3B). Finally, no higher reporting frequency was observed for Oxford–AstraZeneca vaccine compared to Janssen vaccine for all impaired glucose metabolism groups (Figure S3C).

### 2.4. Reporting Rate

The highest reporting rate per 100,000 was observed for Oxford–AstraZeneca vaccine (1.87; 95% CI 1.77–1.97), followed by Moderna vaccine (1.04; 95% CI 0.96–1.12), Janssen vaccine (0.87; 95% CI 0.74–1.01), and Pfizer–BioNTech (0.46; 95% CI 0.44–0.47) (Table S5).

## 3. Discussion

Vaccine safety is an important issue during a vaccination program [14]. In the present analysis, we provided for the first time the European reporting frequency of impaired glucose metabolism events among COVID-19 vaccines for the period between January and December 2021. The most frequently reported events were hyperglycemia and hypoglycemia, regardless of the presence of diabetes. Furthermore, mRNA-based vaccines were associated with a higher reporting frequency of type 1 and type 2 diabetes, hyperglycemia, inadequate control of diabetes, and hypoglycemia as compared with viral vector-based vaccines. Although most cases of impaired glucose metabolism events were identified as serious, they typically showed a favorable outcome with a median duration of 2 or 3 days. Generally, the most common side effects reported with COVID-19 vaccines were pain at inoculation site, fatigue, headache, chills, fever, and flu-like symptoms [15]. Changes in blood glucose levels in diabetic or non-diabetic individuals were not reported in previous Phase 3 clinical trials of COVID-19 vaccines. Few cases of glucose impairment in healthy subjects after COVID-19 vaccination were described in cohort studies or post-market randomized controlled trials [16,17,18]. Acute hyperglycemia (fasting plasma glucose > 170 mg/dL) for 2 days was reported as an unusual sign after Pfizer–BioNTech vaccine [16]. Conversely, one severe event of hypoglycemia (glucose levels of 50 mg/dl) occurring after fasting and vigorous exercise was observed in a man who received the 100 µg dose of mRNA-1273 vaccine [17]. However, this event was considered by the investigators as unrelated to the vaccine [17]. Interestingly, the administration of an inactivated SARS-CoV-2 vaccine with a different mechanism of action showed a consistent increase in HbA1c levels in healthy volunteers. HbA1c levels reached a pre-diabetic range after 28 days of vaccination and then gradually decreased within 90 days, maintaining still significantly higher levels than those observed before vaccination [18]. Whether COVID-19 vaccination may cause perturbation of glucose levels in diabetic patients remains controversial. A retrospective analysis of 96 adults (age ≥ 18 years) with type 1 diabetes using a flash glucose monitoring system (FGM) revealed that 59% of individuals experienced major perturbations of glucose control. Specifically, 30% of patients showed a decrease of time in range (TIR) of over 10%, and 10% showed a decrease of over 20% in the 7 days immediately after first COVID-19 vaccination, with no difference between mRNA or viral vector-based vaccines [19]. An analysis of 20 individuals with type 1 diabetes and FGM showed the worst increase in glucose levels after vaccination in elderly patients on oral hypoglycemic medication (metformin and dapagliflozin) and basal–bolus insulin regimen [20]. On the other hand, no significant difference in TIR was found before or after second dose vaccination in 35 subjects with type 1 diabetes using a continuous glucose monitoring (CGM) device [21]. Recently, a retrospective study of 161 individuals with type 1 and type 2 diabetes showed no significant difference in TIR with COVID-19 vaccination [22]. Interestingly, in a sub-analysis of the same study, a deterioration of glucose levels represented by a decrease of TIR and an increase of time above range (TAR) was observed in type 1 diabetes patients [22]. The mechanisms linking COVID-19 vaccines and the alterations of glucose homeostasis may be only hypothesized. SARS-CoV-2 infection has been demonstrated to worsen glucose control in patients with and without diabetes [23,24]. Generally, anti-viral vaccines may cause unstable blood glucose levels, not only as a reaction to the virus but also to the vaccine-related excipients. A case of acute hyperglycemia was also reported after influenza vaccination in a type 2 diabetes patient [25]. Vaccines can activate the immune system and inflammation, which can in turn impair insulin sensitivity and increase blood glucose levels [26]. High levels of cytokines (IL-1, IL-6, IFNγ, and TNFα) can occur in the presence of triggers such as vaccine excipients, the adenoviral vector (for viral vector-based vaccines), or the SARS-CoV-2 spike protein (for mRNA vaccines) and may lead to pancreatic damage and acute hyperglycemia [11,12]. In particular, hypercytokinemia can decrease pancreatic blood flow, alter β-cell activity, and raise oxidative stress, reducing insulin production and insulin sensitivity in tissues [27].

Vaccine-related adverse events vary considerably according to age and sex, with more severe effects occurring in women and younger people [28]. In our analysis, the prevalence of ICSRs was higher in females than males, as reported in previous studies [29,30]. Notably, women present stronger immune responses against pathogens and vaccines but also higher susceptibility to autoimmune diseases [28]. Previous studies have revealed that SARS-CoV-2 infection could trigger autoimmunity and the onset of new diabetes [31,32], but the association between COVID-19 vaccines and autoimmune phenomena remains uncertain. Five cases of Graves’ disease [33,34,35] and one case of conversion of pre-existing type 2 diabetes into type 1 diabetes (testified by positivity for GAD65 antibodies and low C-peptide levels) have been reported following the Pfizer vaccine administration [35]. The main mechanisms by which the COVID-19 vaccine may trigger the autoimmune response include molecular mimicry, the production of specific pro-inflammatory cytokines, and the role of certain vaccine adjuvants [14]. However, the existence of a causal relationship between COVID-19 vaccines and the development of an autoimmune response remains to be clarified. The overall prevalence of ICSRs in our analysis is low, with a higher reporting rate for Oxford–AstraZeneca vaccine (reporting rate 1.87). This low frequency was also observed in a previous pharmacovigilance analysis of data from VigiBase (WHO Collaborating Centre for International Drug Monitoring). This analysis for COVID-19 vaccines showed a total of 1464 ICSRs related to hyperglycemia, 1137 to diabetes mellitus, 25 to hyperosmolar hyperglycemic syndrome, and 398 to diabetic ketoacidosis. However, it remains unclear whether the low frequency is due to rarity, a lack of proper vaccine surveillance, and/or under-reporting [13]. Indeed, under-reporting is a primary concern in pharmacovigilance that needs continuous surveillance and proactive stimulation.

In conclusion, mRNA COVID-19 vaccines were associated with an increased reporting frequency of some alterations of glucose homeostasis compared to viral-vector COVID-19 vaccines. Clinicians should be aware of these events to better manage glycemic perturbations and to monitor blood glucose levels in high-risk subjects such as pre-diabetic, diabetic, or patients with a history of COVID-19. Larger nationwide studies are warranted to verify these findings. Two clinical trials are currently ongoing to assess the effect of COVID-19 vaccines on blood glucose levels in patients with diabetes (NCT04923386; NCT05233592) [23,24]. 

### Strengths and Limitations

The present study carries some strengths and limitations. Among the strengths, this study is the first analysis comparing the frequency of impairment of glucose metabolism among the anti-COVID-19 vaccines currently in use. The spontaneous reporting system is an inexpensive and useful tool for the collection and analysis of drug safety data. Indeed, we can detect ADRs not detectable during the pre-marketing phase, such as rare and serious ones, through data from the spontaneous reporting system. We analyzed ICSRs related to anti-COVID-19 vaccines by using a large data source (the EV database) that covers the entire European area. Among limitations, there is the under-reporting and the poor quality of information listed in each ICSR. Under-reporting is a major limitation of spontaneous reporting systems that reduces sensitivity due to underestimation of frequency and impact of a given ADR. Moreover, the incompleteness of information reported in the ICSRs could have affected our results by influencing case selection. Indeed, ICSRs were missing for comorbidities, vaccination dose number, and prior history of SARS-CoV-2 infection.

## 4. Materials and Methods

### 4.1. Data Source

ICSRs with COVID-19 mRNA vaccines and COVID-19 viral vector-based vaccines as suspected vaccines were retrieved from EV, a pharmacovigilance database available at www.adrreports.eu, for the period from 1 January 2021 to 11 December 2021. The EV contains all ICSRs reported by an HCP or a non-HCP to an EU national competent authority or a marketing authorization holder. The EV is supervised by the EMA, and it is a system used for the management and analyses of ICSRs related to both medicines or vaccines authorized for use or being evaluated in clinical trials within the EEA.

### 4.2. ICSRs Selection with Line Listing

ICSRs with COVID-19 mRNA vaccines and COVID-19 viral vector-based vaccines were retrieved by using the line listing function. Pfizer–BioNTech and Moderna vaccines were considered for COVID-19 mRNA vaccines, and Oxford–AstraZeneca and Janssen (Johnson & Johnson) vaccines for COVID-19 viral vector-based vaccines. The Novavax vaccine was not considered because it was authorized later, on 20 December 2021. An ICSR related to events of impaired glucose metabolism was identified by using selected PTs from Standardized Medical Dictionary for Regulatory Activities (MedDRA) queries “Hyperglycaemia/new onset diabetes mellitus” and “Hypoglycaemia”. The selection of PTs was performed by expert diabetologists and is listed in Table S1. MedDRA is a standardized, highly specified medical terminology that allows international sharing of regulatory health information for medical products. MedDRA is structured in five levels, from the most general to the most specific: system organ class (SOC), high level group terms (HLGT), lowest high level terms (HLT), PT, and lowest level terms (LLT) [36,37,38]. An integral part of an MedDRA subscription is represented by Standardised MedDRA queries (SMQs), which are a newly developed tool for retrieving cases of interest from a MedDRA-coded database (such as the pharmacovigilance databases). SMQs contain several terms associated with signs, symptoms, diagnoses, syndromes, and physical or laboratory findings that are associated with a specific medical condition [39]. Finally, to identify the presence of diabetes mellitus at the time of vaccination, ICSRs were classified based on the presence of concomitant anti-diabetic agents. Anti-diabetic agents are shown in Table S2.

### 4.3. Descriptive Analyses

Information on patient characteristics (age and gender), seriousness and outcome of the adverse event, primary source qualification, primary source country for regulatory purposes, and number of ICSRs with concomitant anti-diabetic agents was provided for the type of vaccine. In accordance with the International Council on Harmonization E2D guidelines, we classified the seriousness of an adverse event as life-threatening, results in death, caused/prolonged hospitalization, disabling, determines a congenital anomaly/birth defect, or other medically important condition. In case of more than one criterion of seriousness for an adverse event, we chose for classification the most serious. The outcome of the adverse event was classified as “Recovered/Resolved”, “Recovering/Resolving”, “Recovered/Resolved with Sequelae”, “Not Recovered/Not Resolved”, “Fatal”, and “Unknown”. The outcome with the lower level of resolution was chosen for classification when an adverse event reported two or more different outcomes. The monthly trend of ICSRs related to impaired glucose metabolism events was also provided. Impaired glucose metabolism events were described and analyzed based on the diabetologists’ classification into nine groups (Table S1): “diabetes in pregnancy”, “acute complications of diabetes”, “pre-diabetes”, “type 1 diabetes mellitus”, “type 2 diabetes mellitus”, “high glucose levels”, “diabetes mellitus inadequate control”, “diabetes mellitus not specified”, and “hypoglycaemia”. The median duration of impaired glucose metabolism events and the IQR were reported in days within boxplots for each vaccine. This computation was performed only on ICSRs containing the duration of the event. Boxplots were performed using R (version 3.2.2, R Development Core Team).

### 4.4. Disproportionality Analyses

The ROR, its 95% CI, and the chi-square test were computed to assess the reporting frequency of impaired glucose metabolism groups of mRNA vaccines compared to viral vector-based vaccines: Pfizer–BioNTech vaccine compared to Moderna vaccine and Oxford–AstraZeneca vaccine compared to Janssen vaccine. Sensitivity disproportionality analyses were performed by using only ICSRs with or without concomitant anti-diabetic agents. Sensitivity analyses on ICSRs with concomitant anti-diabetic agents were performed by excluding the groups “pre-diabetes”, “type 1 diabetes mellitus”, “type 2 diabetes mellitus”, and “diabetes mellitus not specified” because we assumed that patients already had diabetes mellitus at the time of vaccination. Similarly, for ICSRs without concomitant anti-diabetic agents, the group “acute complications of diabetes” was not considered because it implied the presence of diabetes mellitus. RORs were carried out for groups that reported ≥3 cases. Data management and analysis were performed with Microsoft Excel 2019 and R (version 3.2.2, R Development Core Team). 

### 4.5. Reporting Rate

The reporting rate for each vaccine was computed by dividing the number of impaired glucose metabolism events with the number of vaccine doses given to people in Europe as of 1 December 2021 per 100,000. The number of vaccine doses given in Europe was retrieved from the EMA website [40].

## Figures and Tables

**Table 1:** Distribution for age, gender, primary source, primary source country, presence of concomitant anti-diabetic agents among Individual Case Safety Reports (ICSRs) reporting at least one event of impaired glucose metabolism and having mRNA or viral vector COVID-19 vaccines as suspected vaccine among those reported in Eudravigilance from 1 January 2021 to 11 December 2021.

Variable	Level	COVID-19 mRNA Vaccines (*n* = 2613)	COVID-19 Viral Vector-Based Vaccines (*n* = 1304)
Age	<18 years (%)	55 (2.10)	8 (0.61)
	18–64 years (%)	1531 (58.60)	876 (67.18)
	>65 years (%)	867 (33.18)	311 (23.85)
	Missing (%)	160 (16.12)	109 (8.36)
Gender	F (%)	1525 (58.36)	767 (58.82)
	M (%)	1055 (40.38)	509 (39.03)
	Missing (%)	33 (1.26)	28 (2.15)
Primary Source	Healthcare Professional (%)	1261 (48.26)	445 (34.13)
	Non-Healthcare Professional (%)	1352 (51.74)	859 (65.87)
Primary Source Country for Regulatory Purposes	European Economic Area (%)	1335 (51.09)	500 (38.34)
	Non-European Economic Area (%)	1278 (48.91)	804 (61.66)
Patients with concomitant anti-diabetic agents	Yes (%)	635 (24.30)	459 (35.20)
	No (%)	1978 (75.70)	845 (64.80)

**Table 2:** Distribution for age, gender, primary source, primary source country, presence of concomitant anti-diabetic agents among Individual Case Safety Reports (ICSRs) reporting at least one event of impaired glucose metabolism and having Pfizer–BioNTech, Moderna, Oxford–AstraZeneca, or Janssen Vaccine as suspected vaccine among those reported in Eudravigilance from 1 January 2021 to 11 December 2021.

Variable	Level	Pfizer–BioNTech Vaccine (*n* = 2027)	Moderna Vaccine (*n* = 586)	Oxford–AstraZeneca Vaccine (*n* = 1163)	Janssen Vaccine (*n* = 141)
Age	<18 years (%)	53 (2.61)	2 (0.34)	8 (0.69)	0 (0.00)
	18–64 years (%)	1199 (59.15)	332 (56.66)	774 (66.55)	102 (71.34)
	>65 years (%)	636 (31.38)	231 (39.42)	278 (23.90)	33 (23.40)
	Missing (%)	139 (6.86)	21 (3.58)	103 (8.86)	6 (4.26)
Gender	F (%)	1194 (58.91)	331 (56.48)	699 (60.10)	68 (48.23)
	M (%)	804 (39.66)	251 (42,83)	437 (37.58)	72 (51.06)
	Missing (%)	29 (1.43)	4 (0.68)	27 (2.32)	1 (0.71)
Primary Source	Healthcare Professional (%)	850 (41.93)	411 (70.14)	364 (31.30)	81 (57.45)
	Non-Healthcare Professional (%)	1177 (58.07)	175 (29.86)	799 (68.70)	60 (42.55)
Primary Source Country for Regulatory Purposes	European Economic Area (%)	1170 (57.72)	165 (28.16)	464 (39.90)	36 (25.53)
	Non-European Economic Area (%)	857 (42.28)	421 (71.84)	699 (60.10)	105 (74.47)
Patients with concomitant anti-diabetic agents	Yes (%)	507 (25.01)	128 (21.84)	424 (36.46)	35 (24.82)
	No (%)	1520 (74.99)	458 (78.16)	739 (63.54)	106 (75.18)

**Table 3:** Seriousness and outcome of impaired glucose metabolism events distributed by mRNA or viral vector-based COVID-19 vaccines and reported in Eudravigilance from 1 January 2021 to 11 December 2021.

Variable	Level	COVID-19 mRNA Vaccines (*n* = 2832)	COVID-19 Viral Vector-Based Vaccines (*n* = 1443)
Seriousness	Caused/Prolonged Hospitalisation (%)	461 (16.28)	181 (12.54)
	Other Medically Important Condition (%)	878 (31.00)	551 (38.18)
	Life Threatening (%)	190 (6.71)	110 (7.62)
	Results in Death (%)	90 (3.18)	39 (2.70)
	Disabling (%)	118 (4.17)	74 (5.13)
	Congenital Anomaly (%)	1 (0.04)	1 (0.07)
	Not Serious (%)	1094 (38.63)	487 (33.75)
Outcome	Recovered/Resolved (%)	765 (27.01)	395 (27.37)
	Recovering/Resolving (%)	390 (13.77)	301 (20.86)
	Not Recovered/Not Resolved (%)	694 (24.51)	410 (28.41)
	Fatal (%)	89 (3.14)	27 (1.87)
	Recovered/Resolved with Sequelae (%)	72 (2.54)	20 (1.39)
	Unknown (%)	822 (29.03)	290 (20.10)

**Table 4:** Seriousness and outcome of impaired glucose metabolism events distributed by type of COVID-19 vaccine and reported in Eudravigilance from 1 January 2021 to 11 December 2021.

Variable	Level	Pfizer–BioNTech Vaccine (*n* = 2194)	Moderna Vaccine (*n* = 638)	Oxford–AstraZeneca Vaccine (*n* = 1286)	Janssen Vaccine (*n* = 157)
Seriousness	Caused/Prolonged Hospitalisation (%)	267 (12.17)	194 (30.41)	129 (10.03)	52 (33.12)
	Other Medically Important Condition (%)	761 (34.69)	117 (18.34)	506 (39.35)	45 (28.66)
	Life Threatening (%)	118 (5.38)	72 (11.29)	89 (6.92)	21 (13.38)
	Results in Death (%)	44 (2.01)	46 (7.21)	23 (1.79)	16 (10.19)
	Disabling (%)	91 (4.15)	27 (4.23)	69 (5.37)	5 (3.18)
	Congenital Anomaly (%)	1 (0.05)	0 (0.00)	1 (0.08)	0 (0.00)
	Not Serious (%)	912 (41.57)	182 (28.53)	469 (36.47)	18 (11.46)
Outcome	Recovered/Resolved (%)	592 (26.98)	173 (27.12)	376 (2.24)	19 (12.10)
	Recovering/Resolving (%)	344 (15.68)	46 (7.21)	286 (22.24)	15 (9.55)
	Not Recovered/Not Resolved (%)	523 (23.84)	171 (26.80)	347 (26.98)	63 (40.13)
	Fatal (%)	43 (1.96)	46 (7.21)	16 (1.24)	11 (7.01)
	Recovered/Resolved with Sequelae (%)	69 (3.14)	3 (0.47)	19 (1.48)	1 (0.64)
	Unknown (%)	623 (28.40)	199 (31.19)	242 (18.82)	48 (30.57)

## Data Availability

Data is contained within the article and Supplementary Materials.

## Supplementary Materials

The following supporting information can be downloaded at: https://www.mdpi.com/article/10.3390/ph15060677/s1, Table S1: List of MedDRA (Medical Dictionary for Regulatory Activities) preferred terms of impaired glucose metabolism classified into event groups; Table S2: List of anti-diabetic agents; Figure S1: Trend of Individual Case Safety Reports (ICSRs) reported in Eudravigilance with at least one event of impaired glucose metabolism for each type of COVID-19 vaccine; Table S3: Total number of adverse events for each type of COVID-19 vaccine up to 11 December 2021; Table S4: Distribution of preferred terms for event group classification; Figure S2: Reporting Odds Ratio (ROR) of impaired glucose metabolism groups comparing COVID-19 mRNA vaccines with COVID-19 viral vector-based vaccines (**A**), Pfizer–BioNTech vaccine with Moderna vaccine (**B**), and Oxford–AstraZeneca vaccine with Janssen vaccine (**C**) in ICSRs with concomitant anti-diabetic agents; Figure S3: Reporting Odds Ratio (ROR) of impaired glucose metabolism groups comparing COVID-19 mRNA vaccines with COVID-19 viral vector-based vaccines (**A**), Pfizer–BioNTech vaccine with Moderna vaccine (**B**), and Oxford–AstraZeneca vaccine with Janssen vaccine (**C**) ICSRs without concomitant anti-diabetic agents; Table S5: Reporting Rate of impaired glucose metabolism event for each COVID-19 vaccine up to 1 December 2021.
