# Supplementary material for: European Safety Analysis of mRNA and Viral Vector COVID-19 Vaccines on Glucose Metabolism Events
Source: Pharmaceuticals (Basel). 2022 May 27;15(6):677. doi: 10.3390/ph15060677 (PMC9229409; doi:10.3390/ph15060677)
Supplement: Supplementary file 1 [file pharmaceuticals-15-00677-s001.zip › pharmaceuticals-1713213-supplementary.pdf]

## SUPPLEMMENTARY MATERIALS

**Table S1.** List of MedDRA (*Medical Dictionary for Regulatory Activities*) preferred terms of impaired glucose metabolism classified into event groups.

| | Event Groups | Preferred terms |
|---|---|---|
| *1.* | *Diabetes in pregnancy* | Diabetes complicating pregnancy<br>Gestational diabetes<br>Glucose tolerance impaired in pregnancy<br>Glycosuria during pregnancy |
| *2.* | *Acute complications of Diabetes* | Ketosis-prone diabetes mellitus<br>Euglycaemic diabetic ketoacidosis<br>Diabetic ketosis<br>Diabetic ketoacidosis<br>Diabetic ketoacidotic hyperglycaemic coma<br>Diabetes with hyperosmolarity<br>Hyperglycaemic seizure<br>Hyperglycaemic unconsciousness |
| *3.* | *Pre-diabetes* | Impaired fasting glucose<br>Glucose tolerance impaired<br>Glucose tolerance impaired<br>Glucose tolerance test abnormal |
| *4.* | *Type 1 diabetes mellitus* | Type 1 diabetes mellitus<br>Anti-GAD antibody positive<br>Anti-IA2 antibody positive<br>Anti-insulin antibody increased<br>Anti-insulin antibody positive<br>Latent autoimmune diabetes in adults<br>Fulminant type 1 diabetes mellitus |
| *5.* | *Type 2 diabetes mellitus* | Type 2 diabetes mellitus<br>Insulin-requiring type 2 diabetes mellitus |
| *6.* | *High glucose level* | Blood glucose abnormal<br>Blood glucose increased<br>Hyperglycaemia<br>Glycosuria<br>Glucose urine present<br>Glycosylated haemoglobin abnormal<br>Glycosylated haemoglobin increased<br>Insulin resistance |
| *7.* | *Diabetes mellitus inadequate control* | Diabetes mellitus inadequate control<br>Insulin therapy<br>Insulin resistant diabetes |
| *8.* | *Diabetes mellitus not specified* | Diabetes mellitus |
| *9.* | *Hypoglycaemia* | Hypoglycaemia<br>Blood glucose decreased<br>Hypoglycaemia unawareness<br>Hypoglycaemic coma<br>Hypoglycaemic encephalopathy<br>Hypoglycaemic seizure<br>Postprandial hypoglycaemia<br>Hypoglycaemic unconsciousness |

**Table S2.** List of anti-diabetic agents.

| Drug Class | ATC code | Active ingredients |
|---|---|---|
| **Biguanides** | A10BA01<br>A10BA02<br>A10BA03 | phenformin<br>metformin<br>buformin |
| **Sulfonylureas** | A10BB01<br>A10BB02<br>A10BB03<br>A10BB04<br>A10BB05<br>A10BB06<br>A10BB07<br>A10BB08<br>A10BB09<br>A10BB10<br>A10BB11<br>A10BB12<br>A10BB31 | glibenclamide<br>chlorpropamide<br>tolbutamide<br>glibornuride<br>tolazamide<br>carbutamide<br>glipizide<br>gliquidone<br>gliclazide<br>metahexamide<br>glisoxepide<br>glimepiride<br>acetohexamide |
| **Sulfonamides (heterocyclic)** | A10BC01 | glymidine |
| **Alpha glucosidase inhibitors** | A10BF01 | acarbose |
| **Thiazolidinediones** | A10BG01<br>A10BG02<br>A10BG03<br>A10BG04 | troglitazone<br>rosiglitazone<br>pioglitazone<br>lobeglitazone |
| **Dipeptidyl peptidase 4 (DPP-4) inhibitors** | A10BH01<br>A10BH02<br>A10BH03<br>A10BH04<br>A10BH05<br>A10BH06<br>A10BH07<br>A10BH08<br>A10BH51<br>A10BH52 | sitagliptin<br>vildagliptin<br>saxagliptin<br>alogliptin<br>linagliptin<br>gemigliptin<br>evogliptin<br>teneligliptin<br>sitagliptin and simvastatin<br>gemigliptin and rosuvastatin |

| | | |
|---|---|---|
| **Glucagon-like peptide-1 (GLP-1) analogues** | A10BJ01<br>A10BJ02<br>A10BJ03<br>A10BJ04<br>A10BJ05<br>A10BJ06<br>A10BJ07 | exenatide<br>liraglutide<br>lixisenatide<br>albiglutide<br>dulaglutide<br>semaglutide<br>beinaglutide |
| **Sodium-glucose co-transporter 2 (SGLT2) inhibitors** | A10BK01<br>A10BK02<br>A10BK03<br>A10BK04<br>A10BK05<br>A10BK06<br>A10BK07 | dapagliflozin<br>canagliflozin<br>empagliflozin<br>ertugliflozin<br>ipragliflozin<br>sotagliflozin<br>luseogliflozin |
| **Other blood glucose lowering drugs, excl. insulins** | A10BX01<br>A10BX02<br>A10BX03<br>A10BX05<br>A10BX06<br>A10BX08<br>A10BX15 | guar gum<br>repaglinide<br>nateglinide<br>pramlintide<br>benfluorex<br>mitiglinide<br>imeglimin |
| **Insulins and analogues** | A10AB<br>A10AC<br>A10AD<br>A10AE<br>A10AF | Insulins and analogues for injection, fast-acting<br>Insulins and analogues for injection, intermediate-acting<br>Insulins and analogues for injection, intermediate- or long-acting combined with fast-acting<br>Insulins and analogues for injection, long-acting<br>Insulins and analogues for inhalation |

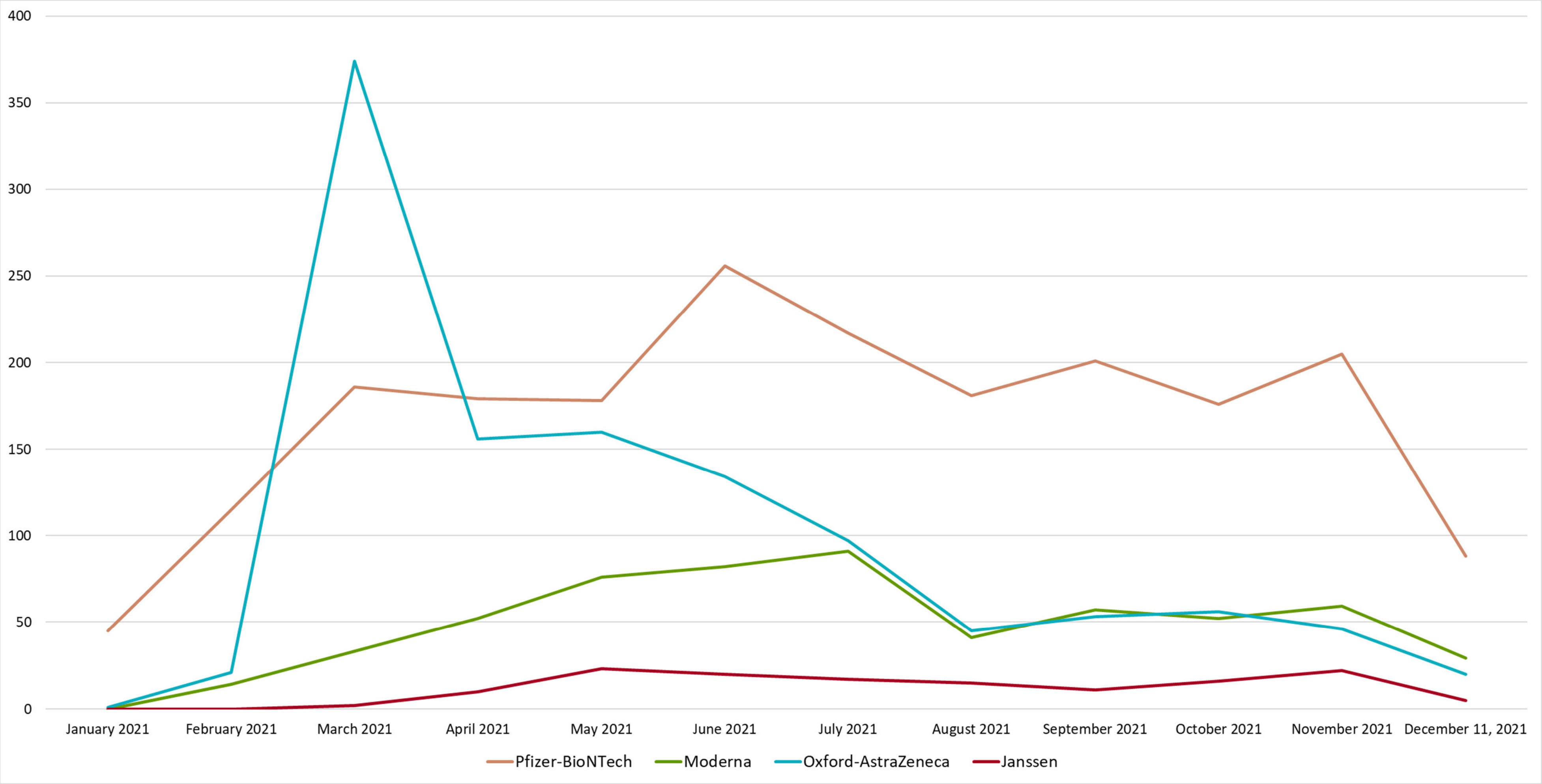


**Figure S1**. Trend of Individual Case Safety Reports (ICSRs) reported in Eudravigilance with at least one event of impaired glucose metabolism for each type of COVID-19 vaccine.

**Table S3.** Total number of adverse events for each type of COVID-19 vaccine up to December 11th, 2021.

| COVID-19 Vaccine | Number of adverse events |
|---|---|
| Pfizer-BioNTech | 2,080,965 |
| Moderna | 690,174 |
| Oxford-AstraZeneca | 1,670,072 |
| Janssen | 178,943 |

**Table S4.** Distribution of preferred terms for event group classification.

| Event Groups | Preferred terms | Pfizer Vaccine (n=2194) | Moderna Vaccine (n=638) | AstraZeneca Vaccine (n=1286) | Janssen Vaccine (n=157) |
|---|---|---|---|---|---|
| Diabetes in pregnancy | Gestational diabetes | 9 (0.41) | 8 (1.25) | 9 (0.70) | 0 (0.00) |
| | Glucose tolerance impaired in pregnancy | 1 (0.05) | 0 (0.00) | 0 (0.00) | 0 (0.00) |
| | Total | 10 (0.46) | 8 (1.25) | 9 (0.70) | 0 (0.00) |
| Acute complications of Diabetes | Euglycaemic diabetic ketoacidosis | 0 (0.00) | 0 (0.00) | 2 (0.16) | 0 (0.00) |
| | Diabetic ketosis | 1 (0.05) | 0 (0.00) | 6 (0.47) | 0 (0.00) |
| | Diabetic ketoacidosis | 58 (2.64) | 63 (9.87) | 55 (4.28) | 7 (4.46) |
| | Diabetic ketoacidotic hyperglycaemic coma | 1 (0.05) | 0 (0.00) | 0 (0.00) | 0 (0.00) |
| | Total | 60 (2.73) | 63 (9.87) | 63 (4.90) | 7 (4.46) |
| Pre-diabetes | Impaired fasting glucose | 5 (0.23) | 0 (0.00) | 0 (0.00) | 0 (0.00) |
| | Glucose tolerance impaired | 9 (0.41) | 5 (0.78) | 4 (0.31) | 0 (0.00) |
| | Glucose tolerance test abnormal | 0 (0.00) | 0 (0.00) | 1 (0.08) | 0 (0.00) |

| | | | | | |
|---|---|---|---|---|---|
| | Total | 14 (0.64) | 5 (0.78) | 5 (0.39) | 0 (0.00) |
| Type 1 diabetes mellitus | Type 1 diabetes mellitus | 98 (4.47) | 24 (3.76) | 38 (2.95) | 7 (4.46) |
| | Anti-GAD antibody positive | 2 (0.09) | 0 (0.00) | 0 (0.00) | 0 (0.00) |
| | Latent autoimmune diabetes in adults | 0 (0.00) | 1 (0.16) | 0 (0.00) | 0 (0.00) |
| | Fulminant type 1 diabetes mellitus | 3 (0.14) | 0 (0.00) | 0 (0.00) | 0 (0.00) |
| | Total | 103 (4.69) | 25 (3.92) | 38 (2.95) | 7 (4.46) |
| Type 2 diabetes mellitus | Type 2 diabetes mellitus | 40 (1.82) | 28 (4.39) | 19 (1.48) | 7 (4.46) |
| | Insulin-requiring type 2 diabetes mellitus | 1 (0.05) | 2 (0.31) | 0 (0.00) | 1 (0.64) |
| | Total | 41 (1.87) | 30 (4.70) | 19 (1.48) | 8 (5.10) |
| High glucose level | Blood glucose abnormal | 62 (2.83) | 11 (1.72) | 37 (2.88) | 6 (3.82) |
| | Blood glucose increased | 581 (26.48) | 123 (19.28) | 363 (28.23) | 55 (35.03) |
| | Hyperglycaemia | 321 (14.63) | 106 (16.61) | 232 (18.04) | 13 (8.28) |
| | Glycosuria | 0 (0.00) | 0 (0.00) | 1 (0.08) | 0 (0.00) |
| | Glycosylated haemoglobin abnormal | 1 (0.05) | 0 (0.00) | 0 (0.00) | 1 (0.64) |
| | Glycosylated haemoglobin increased | 46 (2.10) | 5 (0.78) | 6 (0.47) | 7 (4.46) |
| | Insulin resistance | 17 (0.77) | 4 (0.63) | 14 (1.09) | 0 (0.00) |
| | Total | 1028 (46.86) | 249 (39.03) | 653 (50.78) | 82 (52.23) |
| Diabetes mellitus inadequate control | Diabetes mellitus inadequate control | 147 (6.70) | 42 (6.58) | 75 (5.83) | 4 (2.55) |
| | Insulin therapy | 1 (0.05) | 1 (0.16) | 0 (0.00) | 0 (0.00) |
| | Insulin resistant diabetes | 2 (0.09) | 2 (0.31) | 1 (0.08) | 0 (0.00) |

| | | | | | |
|---|---|---|---|---|---|
| | Total | 150 (6.84) | 45 (7.05) | 76 (5.91) | 4 (2.55) |
| Diabetes mellitus not specified | Diabetes mellitus | 233 (10.62) | 93 (14.58) | (170 (13.22) | 22 (14.01) |
| Hypoglycaemia | Hypoglycaemia | 355 (16.18) | 82 (12.85) | 172 (13.37) | 12 (7.64) |
| | Blood glucose decreased | 189 (8.61) | 34 (5.33) | 71 (5.52) | 15 (9.55) |
| | Hypoglycaemia unawareness | 1 (0.05) | 0 (0.00) | 3 (0.23) | 0 (0.00) |
| | Hypoglycaemic coma | 3 (0.14) | 1 (0.16) | 2 (0.16) | 0 (0.00) |
| | Hypoglycaemic seizure | 1 (0.05) | 1 (0.16) | 0 (0.00) | 0 (0.00) |
| | Postprandial hypoglycaemia | 2 (0.09) | 1 (0.16) | 2 (0.16) | 0 (0.00) |
| | Hypoglycaemic unconsciousness | 4 (0.18) | 1 (0.16) | 2 (0.16) | 0 (0.00) |
| | Total | 555 (25.30) | 120 (18.81) | 252 (19.60) | 27 (17.20) |

**A**

| Event Group | | ROR | Lower 95%CI | Upper 95%CI | p-value |
|---|---|---|---|---|---|
| Acute Complications of Diabetes | 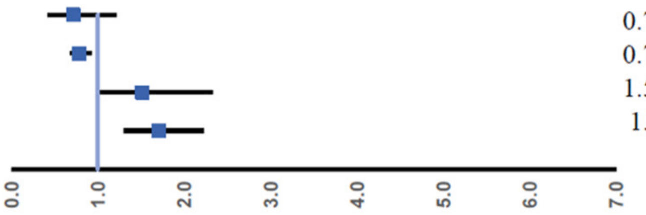 | 0.71 | 0.42 | 1.21 | 0.203 |
| High Glucose Level | | 0.78 | 0.68 | 0.92 | 0.001 |
| Diabetes Mellitus inadequate control | | 1.51 | 0.98 | 2.33 | 0.06 |
| Hypoglycemia | | 1.7 | 1.3 | 2.22 | <0,001 |



**B**

| Event Group | | ROR | Lower 95%CI | Upper 95%CI | p-value |
|---|---|---|---|---|---|
| Acute Complications of Diabetes | 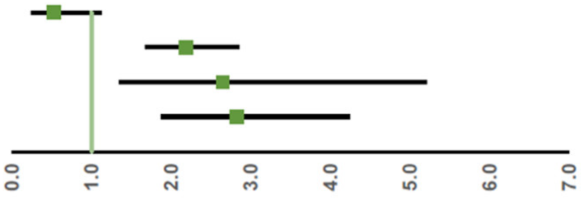 | 0.53 | 0.25 | 1.12 | 0.092 |
| High Glucose Level | | 2.19 | 1.68 | 2.85 | <0.001 |
| Diabetes Mellitus inadequate control | | 2.65 | 1.35 | 5.21 | 0.003 |
| Hypoglycemia | | 2.82 | 1.88 | 4.25 | <0.001 |



**C**

| Event Group | | ROR | Lower 95%CI | Upper 95%CI | p-value |
|---|---|---|---|---|---|
| Acute Complications of Diabetes | 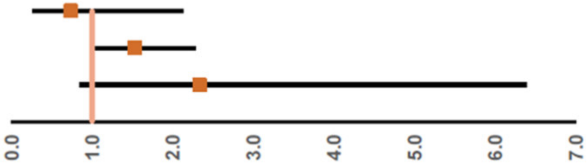 | 0.73 | 0.25 | 2.12 | 0.565 |
| High Glucose Level | | 1.53 | 1.02 | 2.28 | 0.037 |
| Hypoglycemia | | 2.33 | 0.85 | 6.38 | 0.090 |



**Figure S2.** Reporting Odds Ratio (ROR) of impaired glucose metabolism groups comparing COVID-19 mRNA vaccines with COVID-19 viral vector-based vaccines (A), Pfizer-BioNTech vaccine with Moderna vaccine (B), and Oxford-AstraZeneca vaccine with Janssen vaccine (C) in ICSRs with concomitant anti-diabetic agents.

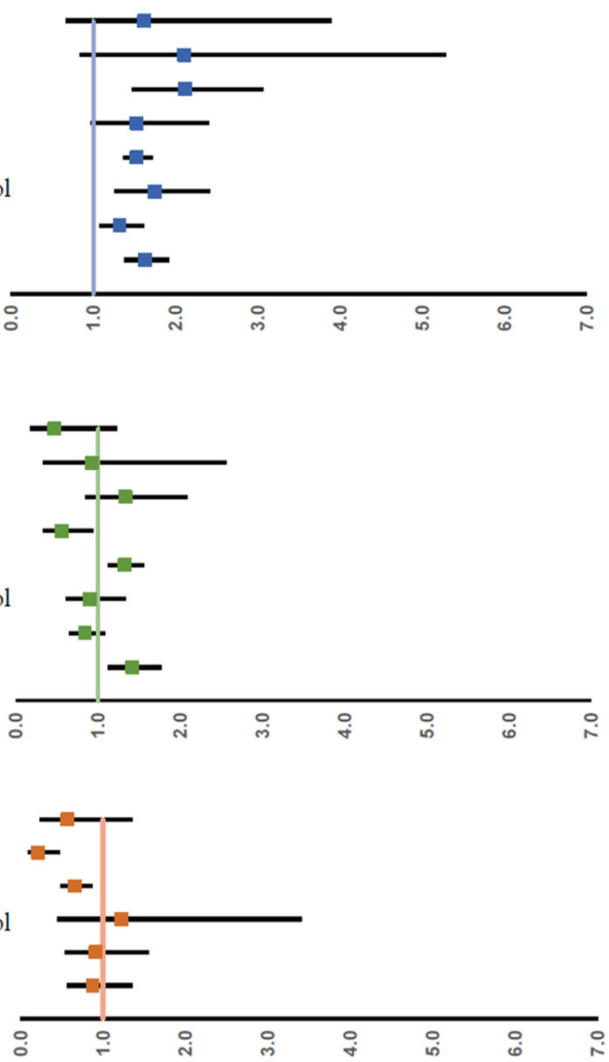


**A**

| Event Group | ROR | Lower 95%CI | Upper 95%CI | p-value |
|---|---|---|---|---|
| Diabetes in Pregnancy | 1.62 | 0.67 | 3.9 | 0.279 |
| Pre-diabetes | 2.11 | 0.84 | 5.29 | 0.103 |
| Type 1 Diabetes Mellitus | 2.12 | 1.47 | 3.06 | <0,001 |
| Type 2 Diabetes Mellitus | 1.53 | 0.97 | 2.41 | 0.063 |
| High Glucose Level | 1.53 | 1.36 | 1.72 | <0,001 |
| Diabetes Mellitus inadequate control | 1.75 | 1.26 | 2.42 | 0.001 |
| Diabetes Mellitus Not Specified | 1.32 | 1.08 | 1.62 | 0.006 |
| Hypoglycemia | 1.63 | 1.38 | 1.92 | <0,001 |

**B**

| Event Group | ROR | Lower 95%CI | Upper 95%CI | p-value |
|---|---|---|---|---|
| Diabetes in Pregnancy | 0.47 | 0.18 | 1.24 | 0.117 |
| Pre-diabetes | 0.92 | 0.33 | 2.56 | 0.877 |
| Type 1 Diabetes Mellitus | 1.33 | 0.85 | 2.08 | 0.208 |
| Type 2 Diabetes Mellitus | 0.56 | 0.33 | 0.94 | 0.025 |
| High Glucose Level | 1.32 | 1.12 | 1.56 | 0.001 |
| Diabetes Mellitus inadequate control | 0.90 | 0.61 | 1.33 | 0.607 |
| Diabetes Mellitus Not Specified | 0.84 | 0.65 | 1.09 | 0.186 |
| Hypoglycemia | 1.42 | 1.13 | 1.78 | 0.002 |

**C**

| Event Group | ROR | Lower 95%CI | Upper 95%CI | p-value |
|---|---|---|---|---|
| Type 1 Diabetes Mellitus | 0.57 | 0.24 | 1.36 | 0.200 |
| Type 2 Diabetes Mellitus | 0.21 | 0.10 | 0.48 | <0.001 |
| High Glucose Level | 0.65 | 0.49 | 0.87 | 0.003 |
| Diabetes Mellitus inadequate control | 1.23 | 0.44 | 3.41 | 0.693 |
| Diabetes Mellitus Not Specified | 0.91 | 0.53 | 1.56 | 0.734 |
| Hypoglycemia | 0.87 | 0.56 | 1.36 | 0.550 |

**Figure S3.** Reporting Odds Ratio (ROR) of impaired glucose metabolism groups comparing COVID-19 mRNA vaccines with COVID-19 viral vector-based vaccines (A), Pfizer-BioNTech vaccine with Moderna vaccine (B), and Oxford-AstraZeneca vaccine with Janssen vaccine (C) ICSRs without concomitant anti-diabetic agents.

**Table S5**. Reporting Rate of impaired glucose metabolism event for each COVID-19 vaccine up to December 1st, 2021.

| | **Vaccine doses given to people in the EU/EEA** | **Reporting Rate/100,000** | **95% Confidence Interval** |
|---|---|---|---|
| Pfizer-BioNTech | 479,000,000 | 0.46 | 0.44-0.47 |
| Moderna | 61,600,000 | 1.04 | 0.96-1.12 |
| Oxford-AstraZeneca | 68,800,000 | 1.87 | 1.77-1.97 |
| Janssen | 18,100,000 | 0.87 | 0.74-1.01 |
